# Determinants of Overall and Progression-Free Survival of Nigerian Patients with Philadelphia-Positive Chronic Myeloid Leukemia

**DOI:** 10.1155/2015/908708

**Published:** 2015-09-07

**Authors:** Anthony A. Oyekunle, Rahman A. Bolarinwa, Adesola T. Oyelese, Lateef Salawu, Muheez A. Durosinmi

**Affiliations:** ^1^Department of Hematology and Immunology, Obafemi Awolowo University, Ile-Ife 234-220005, Nigeria; ^2^Department of Hematology and Blood Transfusion, Obafemi Awolowo University Teaching Hospital Complex, Ile-Ife 234-220005, Nigeria

## Abstract

*Objective*. The tyrosine kinase inhibitors have markedly changed the disease course for patients with Ph^+^ and/or *BCR-ABL1* ^+^ chronic myeloid leukemia (CML). This study was embarked upon to assess the long-term effects of imatinib therapy on survival in adult Nigerian patients with CML. *Methods*. All adult patients on imatinib (400–600 mg) seen from July 2003 to December 2010 were assessed. Male/female distribution was 171/101, with a median age of 38 (range, 20–75) years. Overall survival (OS) and progression-free survival (PFS) were determined using the Kaplan-Meier techniques. *Results*. Of all the 272 patients, 205 were in chronic phase, 54 in accelerated phase, and five in blastic phase, at commencement of imatinib. As at December 2010, 222 were alive. OS at 1 and 5 years was 94% and 63%, while PFS was 89% and 54%, respectively. Similarly, amongst the 205 patients in chronic phase, OS at 1 and 5 years was 97% and 68%, while PFS was 92% and 57%. *Conclusion*. Imatinib's place as first-line therapy in the treatment of CML has further been reinforced in our patients, with improved survival and reduced morbidity, comparable with outcomes in other populations.

## 1. Introduction

Chronic myeloid leukemia (CML) is a myeloproliferative neoplasm caused by the* BCR-ABL1*, a chimeric gene generated as a result of a reciprocal translocation [t(9;22)(q34;q11)], cytogenetically visible as the Philadelphia chromosome (Ph) that places sequences from the* ABL* gene from chromosome 9 downstream of the* BCR* gene on chromosome 22 [1, 2]. CML occurs with an incidence of one to two cases per 100,000 people per year and accounts for 15% of adult leukemias [3]. CML may affect any age group; the peak incidence is between 40 and 60 years with the median age at diagnosis being 53 years in the Western world [4]. However, patients with CML in Nigeria and other African countries, with similar demographic pattern, have a median age of about 38 years [5–8]. Clinically, CML is a bi- or triphasic disease. The chronic phase (present at diagnosis in approximately 85% of patients) is easily controlled with conventional chemotherapy, followed by an unstable accelerated phase and terminating in a blastic phase [9]. Until the 1980s, CML was regarded as incurable and thus inexorably fatal, with effective treatment limited to a minority of patients [10]. The treatment of CML has greatly evolved through the years, with the use of chemotherapeutic agents like interferon (IFN), cytarabine, hydroxyurea, and in some stem cell transplantation being the modality of treatment. However, the discovery of the tyrosine kinase inhibitors, the first being imatinib, which has shown significant activity in all phases of the disease, has altered the course of the disease drastically [11–13]. Imatinib is now recognized as the first-line drug in the treatment of CML, especially in patients in the chronic phase [12, 14, 15]. CML patients in Nigeria, since 2003, like in some other African counties have continued to access imatinib freely through the collaborative efforts of Novartis Pharmaceutical, Axios International, and The Max Foundation. This review is intended to take a look at the long-term survival outcomes of patients with Philadelphia chromosome or* BCR-ABL1*-positive CML on imatinib over a 7-year period.

## 2. Patients and Methods

The study was carried out in accordance with the ethical standards of our institutional ethics review board. All patients were treated according to the Helsinki Declaration of 1975, as revised in Edinburgh 2000. All patients reviewed in this study were originally part of a prospective cohort for the clinical postapproval use of imatinib in Nigeria, and all gave written informed consent after appropriate counselling. All 272 Philadelphia chromosome and/or* BCR-ABL1*-positive CML patients enrolled under the Nigerian arm of the GIPAP programme and receiving imatinib since July 2003 to December 2010 were reviewed. Clinical and haematological parameters considered included organomegaly, complete blood counts, and percentage peripheral blasts. The interval between diagnosis and commencement of imatinib was noted and ranged from 0 to 1673 days. Unfortunately, access to cytogenetic and molecular testing in Nigeria at the time of this study was rather limited, and a significant number of these patients did not take these follow-up tests at the prescribed time. Consequently, molecular testing data was excluded from this study.

Collection of data was in MS Excel spreadsheets (Microsoft 2007, USA) and statistical analysis was done using SPSS 17 package (SPSS Inc., 2008, USA). Data cleaning and validation was done in MS Excel, where it was examined for accuracy, correctness, and consistency and cleaned appropriately.

For the purpose of this analysis, only patients in chronic phase, presenting within 3 months of diagnosis and with complete data, were assessed for risk status using the Sokal and Hasford scoring systems.

Survival analysis was done using the Kaplan-Meier method. For the survival studies, overall survival (OS) was calculated as the time interval between the date of commencement of imatinib and the date of last follow-up (for living patients) or the date of death from any cause. Progression-free survival (PFS) was calculated from the date of starting imatinib to the date of documented disease progression to accelerated or blastic phase or to the date of death, whichever was earlier.

## 3. Results 

### 3.1. Survival Outcomes for All 272 Patients

Over the period of review, 272 patients were enrolled with 205, 54, and 5 of them in chronic, accelerated, and blastic phases, respectively, at diagnosis. Of these, 171 (63%) and 101 (37%) were male and female, respectively. Median age was 38 (range, 20–75) years and median follow-up duration was 81 months.  Table 1 shows a summary of patient characteristics at diagnosis.

Overall survival at 1, 2, and 5 years was 94%, 84%, and 63%, respectively, and correspondingly PFS was 89%, 77%, and 54% (Table 2). At the time of this analysis, median OS and PFS were not yet reached. Using the log-rank statistical test (Mantel-Cox) regression model, with the variables as individual predictors of OS and PFS, revealed that chronic phase disease at diagnosis (*p* = 0.030) and male gender (*p* = 0.041) were associated with significantly better OS (Table 3). Significantly better PFS was associated with the male gender (*p* = 0.005), haematocrit >0.30 (*p* = 0.009), and splenic enlargement of <10 cm at diagnosis (*p* = 0.048). A multivariable Cox regression model using disease phase at diagnosis and gender as predictors for OS revealed that disease phase at diagnosis was the sole significant predictor of OS (*p* = 0.035, OR = 1.995, and 95% CI = 1.049–3.793). Similarly, using gender, hematocrit (>0.30 versus <0.30), and spleen size (>10 versus <10 cm) as predictors for PFS, in a multivariable model, revealed that gender was the sole predictor that attained statistical significance (*p* = 0.019, OR = 1.789, and 95% CI = 1.100–2.911).

### 3.2. Survival Outcomes for 205 Patients in Chronic Phase

Based on the outcomes of the multivariate analysis, we embarked on a more detailed analysis of patients presenting in the chronic phase. Of the 205 patients in chronic phase, 132 were males and 73 were females. Only 78 of these (38%) and 73 (34%), respectively, could be assessed for risk status using the Sokal and Hasford scoring systems (Table 1). After a median follow-up period of 82 months, OS at 1, 2, and 5 years was 97%, 87%, and 68%, respectively, while correspondingly PFS was 92%, 79%, and 57%, respectively. Expectedly, the median OS and PFS for this subgroup (performing better than the whole cohort) were not yet reached at the time of analysis.

Univariate log-rank statistical test of the impact of variables on survival revealed that patients younger than 42 years and those who achieved complete haematologic remission (CHR) within 30 days of commencing imatinib had better OS (*p* = 0.011 and <0.001, resp.) and PFS (*p* = 0.013 and 0.002, resp.). Additionally, patients presenting with anaemia (haematocrit < 0.30) and hepatomegaly also had worse PFS (*p* = 0.022 and 0.035; Table 4). Multivariable Cox regression analysis using CHR within 30 days and age (≤42 versus >42 years) as predictors revealed that both variables remained significantly predictive for OS (*p* < 0.001 and *p* = 0.015, resp.). Similarly, multivariable analysis of CHR within 30 days, age (≤42 versus >42 years), anaemia (haematocrit ≤0.30 versus >0.30), and hepatomegaly as predictors of PFS revealed that the former three variables remained statistically significant (*p* = 0.001, 0.010, and 0.012, resp.).

Only 104 of the 205 patients (50.7%) had carried out cytogenetic studies as frequently as desired and could be assessed for complete cytogenetic response (CCR). Only 52 (50%) of these had achieved CCR at any time during therapy. Log-rank regression analysis of factors predicting attainment of CCR revealed that platelet count (≤250 versus >250 × 10^9^/L) was the only variable that was significantly predictive (*p* = 0.045).

## 4. Discussion

The results presented have further proven the efficacy of imatinib in the treatment of CML in Nigerians. In terms of demographics, the median age of 38 years is comparable with reports from other sub-Saharan populations and significantly differs from populations in the developed parts of the world [8, 16]. The younger age at presentation remains a controversial issue, as researchers are divided on whether this is a mere reflection of the country demographics or a true difference in population genetics or disease biology.

Expectedly, the survival pattern of patients presenting in chronic phase is better than those in more advanced stages of disease. In our previous report on part of this cohort, when we reviewed the first 98 patients, after a 4-year follow-up, the OS at 2 years was 81%, compared to 84% in the present larger cohort after a 7-year follow-up [7]. The 5-year OS of the patients in chronic phase in this study (68%) is also more accurate, given that the actual follow-up already exceeds 60 months. When compared to our historical cohort [6], this 5-year survival estimate is impressive, though it is significantly inferior to what has been reported from studies in several Western countries [17–19]. In 2008, Hochhaus et al. [17] reported a 6-year OS of 76% among a cohort of 532 late CP-CML patients, managed on imatinib after interferon. Similarly, in their review of 1148 CP-CML patients in 2012, Kantarjian et al. [19] reported an 8-year survival of 87%, among those patients managed during the imatinib era (i.e., after 2001). It is also noteworthy that there has been a significant reduction in the duration of time from diagnosis to commencement of imatinib (median, 56 days) in our patients. In our previous review [7], it was 100 days, while in a recent study by Koffi et al. [8], a duration of 282 days was reported.

Though our patients are younger than their counterparts in the developed world, survival was not adversely affected, especially if imatinib was commenced in the chronic phase of disease. Patients' survival has also improved due to this difference. The availability of other ancillary investigative tools particularly the detection of* BCR-ABL1* transcript by PCR can also be said to have impacted positively on when patients are commenced on imatinib.

However, in face of these significant improvements in patient's survival, certain challenges cannot be ignored; our hospital remains the only referral centre in the country for patients to access imatinib freely and many of our patients are domiciled at distant places, making follow-up difficult for some of them. Similarly, access to cytogenetic and molecular testing facilities remains markedly limited. This makes deeper disease monitoring more challenging in this cohort.

In conclusion, imatinib has indeed come to stay as the first-line drug of choice in the management of chronic myeloid leukemia, particularly patients in the chronic phase.

## Figures and Tables

**Table 1:** Summary of clinical parameters of all 272 patients at diagnosis.

Variables	Number	Median	(Range)
Age, years: patient	272	38	(20–75)
Gender: male/female (%)	171/101	(63/37)	
Splenomegaly (cm, BCM)	207	12	(2–38)
Time from diagnosis to imatinib (days)	272	56	(0–2308)
Percent peripheral blasts (%)	112	3	(1–20)
Hematocrit (%)	269	32	(13–49)
WBC (×10^9^/L)	265	83.2	(2.1–710.0)
Platelet count (×10^9^/L)	254	247	(10–1173)

Sokal^*∗*^ score (low/intermediate/high)	78/205	(28/30/20)	
Hasford^*∗*^ score (low/intermediate/high)	73/205	(35/33/5)	

^*∗*^Sokal and Hasford scores were applicable only for the 205 patients in chronic phase, presenting within 3 months of diagnosis.

**(a):** 

Variables^*∗*^	*p* values, for all 272 adult patients			
Survival statistics	OS	SE	PFS	SE
At 1 year	94%	0.016	89%	0.020
At 2 years	84%	0.027	77%	0.030
At 5 years	63%	*0.052*	54%	*0.053*

Median survival	^*∗*^Not reached yet			

**(b):** 

Variables^*∗*^	*p* values, for 205 CP adult patients			
Survival statistics	OS	SE	PFS	SE

At 1 year	97%	0.013	92%	0.020
At 2 years	87%	0.029	79%	0.030
At 5 years	68%	*0.059*	57%	*0.053*

Median survival	^*∗*^Not reached yet			

**Table 3:** Univariate analysis of all 272 patients' characteristics as predictors of overall and progression-free survival.

Parameters at recruitment	Number	OS	PFS
Sex: male/female	171/101	**0.041**	**0.005**
Age (years)			
≤30/>30	68/204	ns	ns
≤38/>38	134/138	ns	ns
≤50/>50	207/65	ns	ns
Hematocrit at diagnosis (%)			
≤30/>30	110/162	0.069	**0.009**
≤35/>35	165/107	0.078	**0.022**
WBC at diagnosis (×10^9^/L)			
≤100/>100	144/128	ns	ns
Platelet count (×10^9^/L)			
≤250/>250	144/128	ns	ns
≤350/>350	187/85	ns	ns

Disease phase at diagnosis			
CP/AP/BP	205/54/5	0.078	ns
CP/Adv	205/59	**0.030**	ns
Spleen (cm, BCM)			
≤10/>10	113/159	ns	**0.048**

*p* values in bold type are significant; those in regular type are close to significance. The variables with better outcomes are written first. OS, overall survival; PFS, progression-free survival; BCM, below the costal margin; ns, not significant; NA, not applicable.

**Table 4:** Univariate analysis of all 205 chronic phase patients' characteristics as predictors of overall and progression-free survival.

Parameters at recruitment	Number	OS	PFS	Number	CCR
Sex: male/female	132/73	ns	0.077	65/39	ns
Age (years)					
≤35/>35	70/135	**0.035**	ns	37/67	ns
≤40/>40	102/103	**0.035**	0.056	54/50	ns
≤42/>42	111/94	**0.011**	**0.013**	60/44	ns
Hematocrit at diagnosis (%)					ns
≤30/>30	76/129	0.098	**0.022**	37/67	ns
≤35/>35	120/85	ns	0.067	58/46	ns
WBC at diagnosis (×10^9^/L)					ns
≤100/>100	144/128	ns	ns	61/43	ns
Platelet count (×10^9^/L)					ns
≤250/>250	144/128	ns	ns	62/42	**0.045**
≤350/>350	187/85	ns	ns		ns

Time-to-imatinib (days)					
>30/≤30	68/137	ns	ns	32/72	ns
>60/≤60	109/96	ns	0.083	49/55	0.083
Time-to-CHR (days)					
≤30/>30	79/126	**<0.001**	**0.002**	16/88	ns
≤90/>90	125/80	**0.010**	**0.049**	50/54	ns
Spleen (cm, BCM)					
Spleen: no/yes	50/155	ns	ns	23/81	ns
≤5/>5	58/147	ns	ns	28/76	ns
≤10/>10	93/112	ns	ns	48/56	ns
Liver (cm, BCM)					
Liver: no/yes	140/65	ns	**0.035**	72/32	ns
≤5/>5	155/50	ns	ns	82/22	ns

*p* values in bold type are significant; those in regular type are close to significance. The variables with better outcomes are written first. OS, overall survival; PFS, progression-free survival; BCM, below the costal margin; ns, not significant; NA, not applicable; CHR, complete haematologic remission; CCR, complete cytogenetic remission.
